# Elbow Motion Trajectory Prediction Using a Multi-Modal Wearable System: A Comparative Analysis of Machine Learning Techniques

**DOI:** 10.3390/s21020498

**Published:** 2021-01-12

**Authors:** Kieran Little, Bobby K Pappachan, Sibo Yang, Bernardo Noronha, Domenico Campolo, Dino Accoto

**Affiliations:** Robotics Research Centre, School of Mechanical and Aerospace Engineering, Nanyang Technological University, Singapore 639798, Singapore; kieran_little@live.co.uk (K.L.); kbobby@ntu.edu.sg (B.K.P.); sibo001@e.ntu.edu.sg (S.Y.); bernardo001@e.ntu.edu.sg (B.N.); d.campolo@ntu.edu.sg (D.C.)

**Keywords:** motion intention detection, assistive robotics, rehabilitation robotics, human-machine interface, machine learning

## Abstract

Motion intention detection is fundamental in the implementation of human-machine interfaces applied to assistive robots. In this paper, multiple machine learning techniques have been explored for creating upper limb motion prediction models, which generally depend on three factors: the signals collected from the user (such as kinematic or physiological), the extracted features and the selected algorithm. We explore the use of different features extracted from various signals when used to train multiple algorithms for the prediction of elbow flexion angle trajectories. The accuracy of the prediction was evaluated based on the mean velocity and peak amplitude of the trajectory, which are sufficient to fully define it. Results show that prediction accuracy when using solely physiological signals is low, however, when kinematic signals are included, it is largely improved. This suggests kinematic signals provide a reliable source of information for predicting elbow trajectories. Different models were trained using 10 algorithms. Regularization algorithms performed well in all conditions, whereas neural networks performed better when the most important features are selected. The extensive analysis provided in this study can be consulted to aid in the development of accurate upper limb motion intention detection models.

## 1. Introduction

Human-machine interfaces (HMIs) are widely used in a range of applications: control of assistive robots, such as robotic prostheses [1,2] and orthoses [3,4,5,6], or teleoperation [7,8]. Motion intention detection (MID) is fundamental for implementing HMIs, as it allows for an efficient, hands-free interaction between the user and the device [9]. Predicting motion intention in the case of upper limb movements, when compared to lower limb, is of particular interest, given that they are the main facilitators of the execution of high-skill and dexterous tasks within the user’s peripersonal space. However, unlike the lower limb, the upper limb typically exhibits far more varied motions during the performance of Activities of Daily Living (ADLs) due to the redundancy embedded in the arm’s kinematics [10] and the kinematics couplings between joint axes [11]. This, coupled with the non-cyclical nature of the upper extremity’s movements, makes MID for the upper limb a challenging problem to solve.

The multiple ways to detect motion intention can be broadly categorised according to the signals collected from the user, the features extracted from the data and the algorithms using said feature data to form predictions.

Physiological signals are one of the most commonly used ones. They are mainly obtained by measuring the electrical activity of the muscles, known as electromyographic (EMG) signals, or the brain, known as electroencefalographic (EEG) signals. Myographic signals are measured using surface electrodes and are typically used in classification systems (also known as discrete control systems), implemented for example as state machines [2], or in continuous control systems (also known as proportional control), where the delivered forces/torques are continuously modulated according to a biological model [12] or using machine learning techniques [13]. Using EMG signals is one of the most common ways of detecting user intention due to the signal being detectable before the actual motion is executed and direct correlation to motion intention [14,15]. However, there are some limitations such as change in skin conductivity due to artefacts or the difference in EMG patterns activation between healthy and pathological subjects [16]. EEG signals can be extracted using an array of electrodes placed on the scalp, which can control robots through processing bioelectric signals [17,18]. Like EMG signals, they are favoured for MID due to their intrinsic relation to human motion and are widely used in brain-computer interfaces as well [3,19,20,21,22]. Their main limitations are the long training periods, poor signal-to-noise ratio and the bulkiness of the sensing system [14]. In some applications, it is helpful to use a combination system of physiological sensors rather than using a single one. Wöhrle et al. [23] proposed a hybrid Field Programmable Gate Array (FPGA)-based system for EMG and EEG-based motion prediction, which can provide high computational speed and low power consumption. Another, less popular, type of physiological signal is the change in the muscles dimensions that occurs due to contraction. Kim et al. [24] developed a sensor that measures the Biceps Brachii (BB) muscle circumference and a model that estimates the elbow torques from the sensor data. Most of the literature that employs this physiological signal uses ultrasound as an acquisition system, which is cumbersome to use due to the associated hardware [25,26]. Nonetheless, these studies highlight the potential of using muscle dimensions variations as a feasible input signal.

Kinematic signals measure changes in joint or segment position (or its derivatives, i.e., velocity, acceleration, etc.). They can be used in ON/OFF systems, where a certain movement triggers a control routine to follow a pre-defined trajectory [27], or use a state-space model to decode the angular velocity of shoulder and motion of elbow [28]. One can also use position data from the initial portion of a movement to classify motion intention [29]. Kinematic data can be useful in motion intention detection if the user still has healthy function of their limbs. However, that is not always the case when considering impaired users, who compose a large part of the intended user pool for most applications. For such users, muscle activity is often decreased and they have difficulty in performing motions. Therefore, kinematic signals can prove unreliable. A common method of measuring kinematic data is using Inertial Measurement Units (IMUs), as they provide global position as well as rotation and orientation data, and they are easy to attach to the body segments. However, IMU signals suffer from bias and noises when integrating the measurements of the accelerometer and gyroscope, which can generate a drift issue [30]. Another method is using adaptive oscillators, which can predict the torque during the elbow flexion [31].

In order to use the signals mentioned above, they must be converted into a form readable by a model/algorithm, i.e., *features* must be extracted. In the case of kinematic signals, this is a straightforward procedure that requires little processing other than filtering, as typical features include position, velocity or acceleration (or their angular equivalent in case of joint data). However, in the case of EMG or EEG, more complex procedures are required, as explained in [32]. In the case of EMG, one can extract time domain features, such as mean absolute value and zero crossing, which are simple to extract and have low-dimensionality but are less effective; frequency domain features, such as power spectrum ratio, considered poor for accurate classification; or time-frequency domain features, such as the continuous wavelet transform, which have high-dimensionality, requiring dimensionality reduction techniques, but are very effective in EMG classification.

To address the limitations presented by the use of the different types of signals, the use of multimodal techniques has seen a growth in popularity. In these techniques, multiple signals are used together so that the shortcomings associated to each signal are compensated by complementing the intention prediction. Park et al. [33] implemented a multimodal interface that uses EMG, bend and pressure sensor data to classify intention to open the hand. They propose adapting the selected signals according to the impairment of the subject, where one mode uses bend sensor data for opening the hand and EMG signals for closing it (for users who can initiate opening motion but have difficulty in maintaining an open hand), and another mode uses EMG data for opening the hand and pressure sensors to close it (for users who have clear EMG pattern for opening the hand but difficulty in closing it). Geng et al. [34] used accelerometers to measure mechanomyographic (MMG) data and predict limb position, whereas EMG data was used to classify upper limb motion. Leeb et al. [35] showed that fusing EMG and EEG data provided a reliable source of data for creating a brain-computer interface able to perform well even under conditions of muscular fatigue.

Once the signals have been collected and their features extracted, the obtained data is input to a predictive algorithm. Generally, one can use either classification or regression algorithms [36]. Classification algorithms try to recognise the pattern of the motion and to classify the intention according to predefined groups of possible outcomes. For example, Gardner et al. [37] used a multimodal approach combining MMG sensors, camera-based visual information and kinematic data to classify hand motion intention according to the type of grasp and the object to be grasped. Classification-based algorithms are useful when the goal is to label the motion, and can have high accuracy due to the discrete nature of the prediction. However, they do not allow for generalisation of motion intention prediction, being limited to a finite number of classes. Regression algorithms can be used to directly predict continuous variables such as position or velocity, which can be a more suitable approach to solve the fundamental problem of prediction of a trajectory of the upper limb.

The aim of this paper is to perform an analysis of kinematic and physiological features for predicting elbow motion intention. We investigate different strategies in terms of used sensors, extracted features and selected algorithms. We employed a multimodal sensing system using EMG, IMU and stretch sensors to extract a large number of features. The features were organised into different groups and their correlation with the motion data was calculated to understand which were more important. Different combinations of features were fed into multiple regression-based models in order to compare the performance of multiple types of regression algorithms.

The rest of this paper is organised as follows. Section 2 outlines the experimental setup for data acquisition, data processing and extracted features, and the selected algorithms to investigate. Section 3 presents the motion prediction results. Section 4 presents discussions and concluding remarks.

## 2. Methods

### 2.1. Experimental Protocol

**Figure 1:**
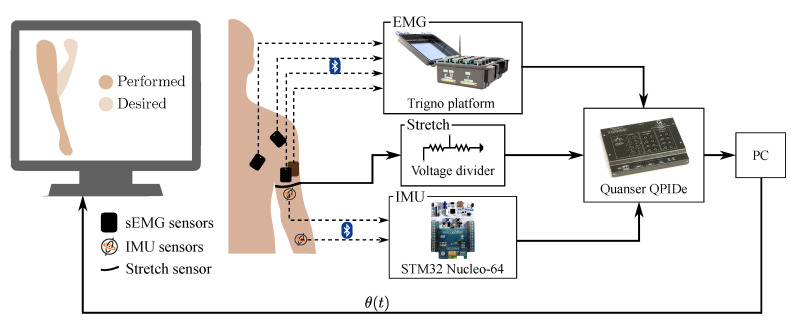
Experimental setup. The subject follows a reference trajectory shown on a computer monitor. The subject’s forearm is simultaneously shown on the screen by reconstructing the elbow angle from the Inertial Measurement Units (IMU) data. The electromyographic (EMG) and IMU data are captured wirelessly and stretch sensor data are captured via a wire. The data are read by the DAQ Quanser QPIDe, which allows the data to be stored on a PC.

Three healthy participants (2M, 1F, age: 26±1 y.o., weight: 60±12 kg, height: 174±10 cm), with no previous history of musculoskeletal problems, were asked to perform a series of elbow flexion motions by following an animation shown on a computer monitor (Figure 1). The motions were limited to the sagittal plane and were performed with forearm pronated 90${}^{\circ }$ (i.e., palm facing centrally), 0${}^{\circ }$ shoulder abduction and 0${}^{\circ }$ shoulder internal/external rotation. All subjects were right-handed, and they all used their dominant side. The subjects were not physically constrained, but instructed to perform the motions according to the above description.

**Figure 2:**
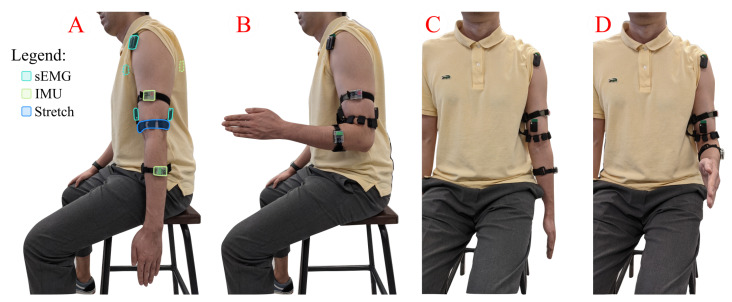
Placement of EMG, IMU and stretch sensors on the user. (**A**,**B**), lateral views of the subject at resting position and when matching the desired elbow angle, respectively; (**C**,**D**) frontal views of the subject at resting position and when matching the desired elbow angle, respectively. The dashed lines represent a sensor that is not visible due to being under the clothes or placed on a side opposite to the current view.

Firstly, IMU, sEMG and stretch sensors were mounted on different locations of the subjects’ upper limb (Figure 2). Then, the subject was shown a target elbow flexion amplitude on a screen and was instructed to flex their elbow to match this position. The elbow angle measured by the IMUs was displayed to give visual feedback to the subject. Each subject completed 300 elbow flexions over 2 sessions with a 10 minute break between sessions to avoid the onset of fatigue. The trajectories varied within a range of ${\left[0,105\right]}^{\circ }$ for the elbow flexion angle and with velocities within the range ${\left[0,92\right]}^{\circ }$/s.

The trajectory of the arm during elbow flexion can be approximated using a minimum-jerk trajectory (MJT). We assumed therefore that the trajectory of the elbow rotation θ(t) when performing flexion movements is of the following form:

(1) $$\begin{array}{cc} \frac{\theta (t)}{\theta_{f}} & =\left[10{\left(t\frac{{\dot{\theta }}_{m}}{\theta_{f}}\right)}^{3}-15{\left(t\frac{{\dot{\theta }}_{m}}{\theta_{f}}\right)}^{4}+6{\left(t\frac{{\dot{\theta }}_{m}}{\theta_{f}}\right)}^{5}\right] \end{array}$$

where $\theta_{f}$ is the peak amplitude of the elbow angle trajectory, ${\dot{\theta }}_{m}$ is the mean angular velocity and $T=\theta_{f}/{\dot{\theta }}_{m}$ is the duration of the movement to reach $\theta_{f}$ from a resting position of $\theta \left(0\right)=0^{\circ }$. From (1), one can see that only two parameters, peak amplitude and mean angular velocity, are needed to fully define the MJT. These two have been used as inference parameters for the predictive model.

### 2.2. Sensors and Data Processing

Three different types of sensors were used to acquire upper limb data: sEMG to measure muscle activity, IMUs to measure elbow flexion angle, and a custom-made stretch sensor to measure changes in muscle volume. All sensors were connected as analog inputs to a real-time data acquisition board (Quanser- QPIDe). Data sampling was performed at a frequency of 167 Hz. All the control scheme was built in Matlab 2014a Simulink.

#### 2.2.1. EMG Sensors

The sEMG sensors (Delsys Trigno) were placed on the muscle belly of the Biceps Brachii (BB), Triceps Brachii (TB), Anteriod Deltoid (AD) and Pectoralis Major (PM) muscles following the SENIAM guidelines [38]. The signals were processed through full-wave rectification followed by low-pass filtering (Butterworth, 4th order, 30 Hz cutoff frequency).

#### 2.2.2. IMU Sensors

The wireless IMU sensors (ST Microelectronics STEVAL-STLKT01V1) are attached on the dorsal side of the forearm and on the dorsal side of the upper arm. The sensors communicate via bluetooth with a development board (STM32 Nucleo-64) equipped with an expansion board (X-NUCLEO-IDB05A1) to allow bluetooth communication, and this board streams the data to the Quanser board. The sensors are then calibrated to a specific body configuration of the user, which is with the shoulder adducted to the trunk and the elbow fully extended. After this calibration is complete, the elbow angle is measured by finding the rotation of the forearm IMU with respect to the upper arm IMU (where a $0^{\circ }$ elbow angle corresponds to the IMUs being aligned). This measurement requires the following assumptions: (1) the IMUs are placed such that the x axis of each sensor is aligned with the axis of the segment to which it is attached and (2) the IMUs do not slide on the skin or move during motion. The raw data from the IMUs were low-pass filtered (Butterworth, 2nd order, 2 Hz cutoff frequency), then differentiated to obtain velocity values and low-pass filtered (Butterworth, 2nd order, 7 Hz cutoff frequency) and finally differentiated again to obtain acceleration data and low-pass filtered again (Butterworth, 2nd order, 9 Hz cutoff frequency).

#### 2.2.3. Stretch Sensor

The stretch sensor (Conductive Rubber Cord Stretch Sensor, Adafruit) was wrapped around the upper arm. This sensor behaves as a variable resistor whose resistance changes according to the extension of the cord. The sensor was connected to a voltage divider circuit where a low-current, low-voltage signal was fed. Changes in the length of the sensor due to changing muscle volume resulted in variations of the read voltage. The data were low-pass filtered (Butterworth, 2th order, 4 Hz cutoff frequency).

The data was segmented in order to isolate the elbow movements, where each segment represents an MJT performed by the subjects.

### 2.3. Feature Extraction

**Figure 3:**
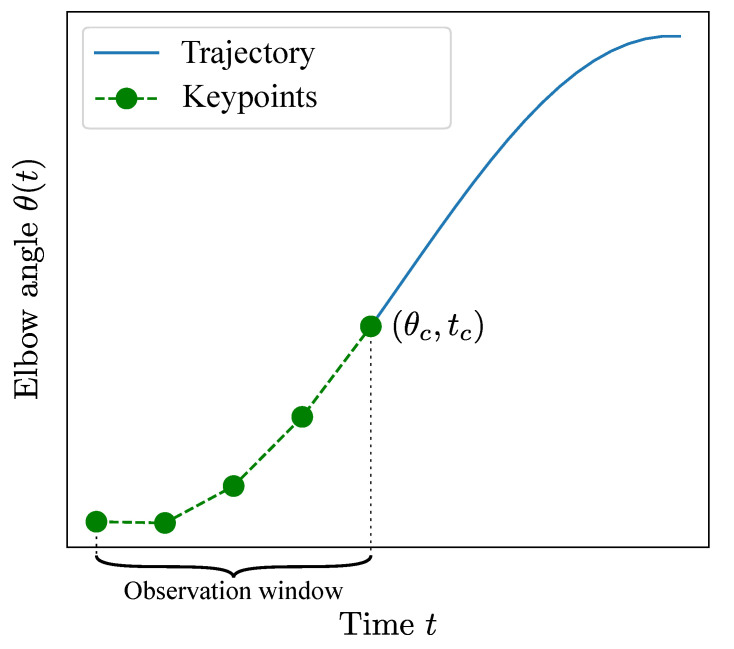
Example of a typical minimum jerk trajectory (MJT) followed by the elbow. The data used for feature extraction is downsampled to a certain number of keypoints which are chosen from within an observation window defined by cutoff angle $\theta_{c}$.

The features used for model training were extracted from the processed and segmented data. Instead of selecting the whole segment for feature extraction, the used data was limited according to 2 factors (Figure 3):

- Observation window (OW). The model is intended to predict motion intention. Therefore, the used data should be limited to a certain time interval at the beginning of the movement. This window is defined between the beginning of the motion ($\theta =0^{\circ }$) and a certain cutoff angle $\theta_{c}$, which is reached at time $t=t_{c}$. The observation window is the time interval $\left[0,t_{c}\right]$, where different $\theta_{c}$ define different OW.
- Keypoints. A certain number $n_{kp}$ of equally distant points was sampled from the selected OW. This was done to avoid overfitting.

**Figure 4:**
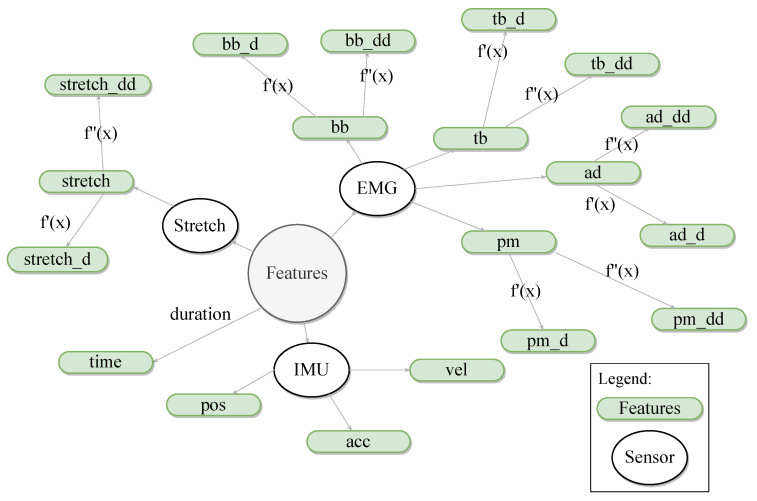
Features extracted from the windowed data, at each keypoint. Features include the EMG, stretch sensor and IMU data and the timestamp at each keypoint, as well as the first and the second derivative of the same data (excluding the timestamp). The features are named as follows: %feature_%derivative, where %feature is the feature name in lowercase letters, and %derivative is the first or second order derivative. For example, “bb” represents the biceps bracchi EMG activity, whereas “bb_d” represents the first derivative of the EMG signal of the BB muscle. bb: biceps bracchi; tb: triceps bracchi; ad: anterior deltoid; pm: pectoralis major; pos: position; vel: velocity; acc: acceleration.

The choice of $\theta_{c}$ and $n_{kp}$ is explained in Section 2.5. The extracted features (Figure 4) consist of the 4 EMG signals, the elbow angle, the stretch sensor signal and the time at each keypoint. Furthermore, the first and second time derivative of the EMG signals, elbow angle and stretch sensor signal are computed at each keypoint and added as features as well, amounting to a total of $\left(12+3+3+1\right)n_{kp}=19n_{kp}$ features.

### 2.4. Cross-Validation

**Figure 5:**
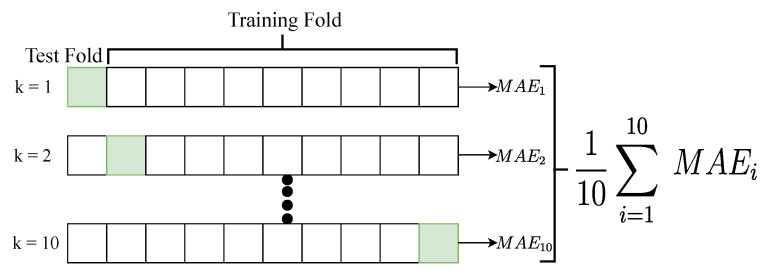
*k*-Fold cross validation.

For evaluating model prediction, *k*-Fold cross validation method is used with *k* value set at 10. Dataset is split into *k*-Fold, were k−1 sets are used for training and the remaining set is used for testing. This process is performed *k* times and the average Mean Absolute Error (MAE) is used to quantify model prediction effectiveness (Figure 5):

(2) $$\mathrm{MAE}=\frac{1}{N}\sum_{i=1}^{N}\left|y_{i}-\hat{y_{i}}\right|$$

### 2.5. Evaluation of Model Performance

The performance of the predictive model was analysed by comparing the predicted values of mean velocity and peak amplitude with the real ones, and computing the MAE according to (2). Performance is affected by four independent factors: $n_{kp}$, OW, selected features and the selected regression algorithms.

$n_{\mathrm{kp}}$: we investigated $n_{kp}$ in the range from 1 to 200.

**OW**: the cut-off angle $\theta_{c}$ took the values of 1,2,3,4 and $5^{\circ }$ to explore different window lengths. We limited the values to $\leq 5^{\circ }$ so that the model prediction is performed for the as close as possible to the onset of the movement.

**Selected features:** the features were organised according to their importance, which was computed using a statistical index known as Mutual Information (MI) [39]. Mutual information quantifies the redundancy between two distributions *X*,*Y* whose joint probability distribution is $P_{XY}\left(x,y\right)$.

(3) $$I\left(X;Y\right)=\sum_{x,y}P_{XY}\left(x,y\right)\log\frac{P_{XY}\left(x,y\right)}{P_{X}\left(x\right)P_{Y}\left(y\right)}$$

**Figure 6:**
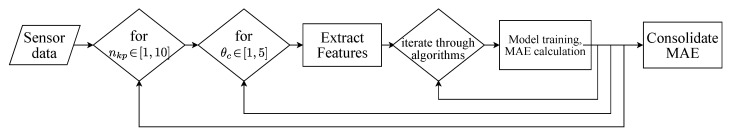
Process flow of Mean Absolute Error (MAE) calculation. This flow is adopted for each set of features and MAE values for each combination is recorded individually.

The MI was calculated between the feature data and the inference parameters, being therefore independent from the used algorithm. MAE for mean velocity and peak amplitude was computed when different sets of features were used: all features, Physiological features only, Kinematic features only, EMG only, Stretch only, IMU+EMG and IMU+stretch. For each set of features the process flow of MAE calculation can be understood from the flowchart in Figure 6.

**Regression algorithms:** Machine learning algorithms are broadly classified as regression and classification. Since the two inference parameters we are interested in are continuous variables and not categorical, the analysis performed in this study focuses on the use of regression algorithms. Ten algorithms based on four different working principles were selected for performance comparison: Ridge, Lasso and Elastic net (based on regularized Least Squares method), Decision Trees, Random Forest, Extra Trees, AdaBoost and Gradient Boosting (based on Decision Trees method), Support Vector Regression (SVR) (based on support vector machines) and Multiple Layer Perceptron (MLP) (based on neural networks).

We are interested to find the best combination of features and algorithms to maximise the accuracy of our inference parameters. The first step was to reduce the complexity of the analysis by selecting the $n_{kp}$ and OW to optimise the performance.

## 3. Results

### 3.1. OW and $n_{kp}$ Variation

**Figure 7:**
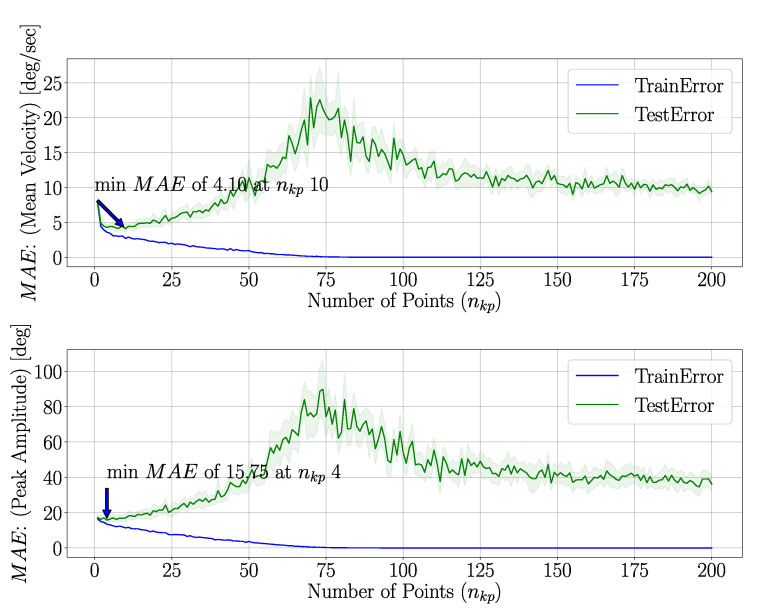
MAE of mean velocity (**top**) and peak amplitude (**bottom**) with changing $n_{kp}$. Training error converges at approximately $n_{kp}=125$ and testing error is lowest below 20 points. All features are used here for model training and MAE calculation.

The intention detection model was trained using a wide range of different $n_{kp}$ to identify the optimum number of samples that can be used to represent a trajectory. As seen in Figure 7, the MAE values are lowest when the number of points is lower for smaller $n_{kp}$. With increasing, $n_{kp}$ the training error plateaus after point number 75. This characteristic shows that the model is prone to over fitting when $n_{kp}$ is larger. Therefore, subsequent analysis is performed with $n_{kp}$ limited to a maximum of 10 points.

**Figure 8:**
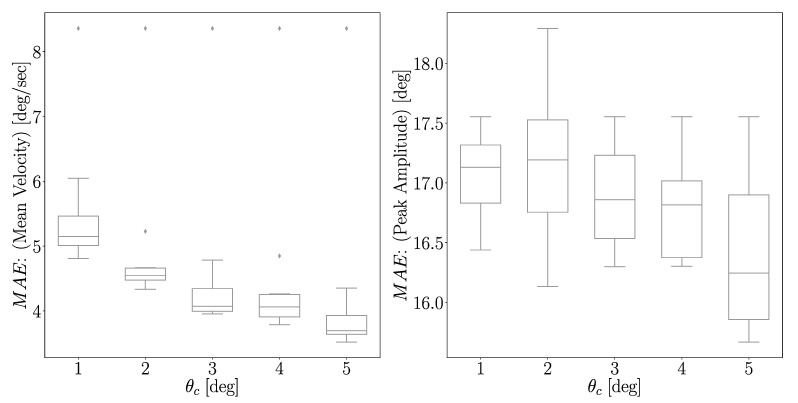
Distribution of MAE of mean velocity and peak amplitude for different $n_{kp}$ and $\theta_{c}$. For a given $\theta_{c}$, the MAE values are calculated when $n_{kp}\in \left[1,200\right]$, and their distribution is plotted. It is clear that MAE values are lower when $\theta_{c}$ increases.

It can be observed that lower MAE values can be attributed to higher OW (Figure 8). In all experiments performed, it was observed that this trend of lower MAE with higher OW persisted. For the mean velocity, the highest observed error was 5.15 deg/s for $\theta_{c}=1^{\circ }$ and the lowest was 3.70 deg/s for $\theta_{c}=5^{\circ }$. The errors for $\theta_{c}=3,4^{\circ }$ were approximately the same, taking the values of 4.07 and 4.06 deg/s, respectively. In the case of peak amplitude, the largest error was 17.19${}^{\circ }$ for $\theta_{c}=1^{\circ }$ and the lowest was 16.24${}^{\circ }$ for $\theta_{c}=5^{\circ }$.

### 3.2. Feature Selection

**Figure 9:**
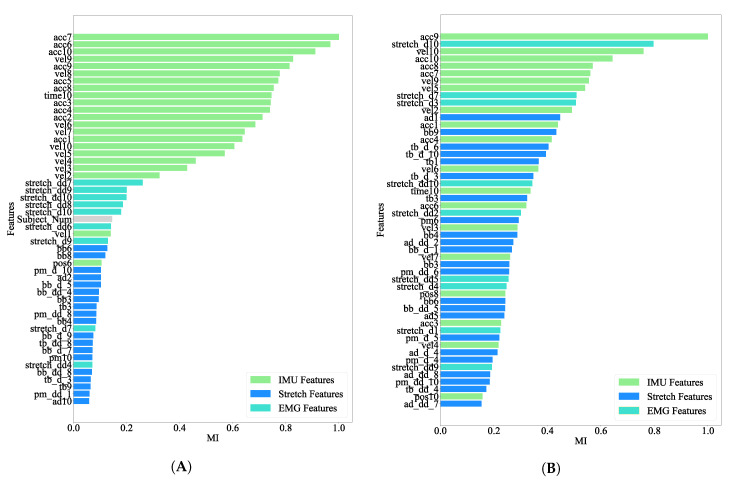
Ranked Mutual Information (MI) of features with respect to the inferred parameters (**A**) mean velocity and (**B**) peak amplitude. For MI calculation the data used is of *n_kp_* at 10, *θ_c_* at 5 and using all available features.Features are color coded as green, blue and turquoise to denote IMU, Stretch and EMG features respectively.

**Figure 10:**
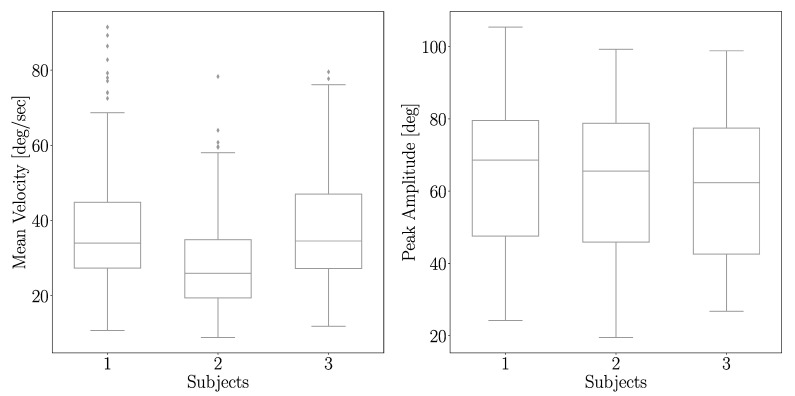
Distribution of inference parameters with respect to subjects. Mean velocity of subject 2 is comparatively lower. In the case of peak amplitude, this trend is not seen.

Model parameter tuning using a high-dimensional feature space can render model coefficients to sub-optimal characteristics. To avoid this, sound theoretical considerations deduced from ground truth or other statistical variance analysis methods are generally used for dimensionality reduction (e.g., PCA, LDA). The MI score helps to identify the relevance of the model training inputs in the larger contextual feature space. MI scores that relate our selected features to the mean velocity and peak amplitude are shown in Figure 9A,B, respectively. As observed from the MI bar-plots, kinematic features generated from the IMU sensor have higher relevance compared with other features while predicting both the mean velocity and peak amplitude. The MAE for peak velocity and mean amplitude when considering all features or only the top 20% most important features (according to the MI score) was calculated (Table 1). One can see that the MAE decreases when only the top 20% most important features are used. A variance analysis of inference parameters between each of the subjects showed results as in Figure 10. Significant change in variance is not observed which indicates that the data is not biased with respect to any of the subjects involved in the experiment.

### 3.3. Comparison of Algorithms

**Figure 11:**
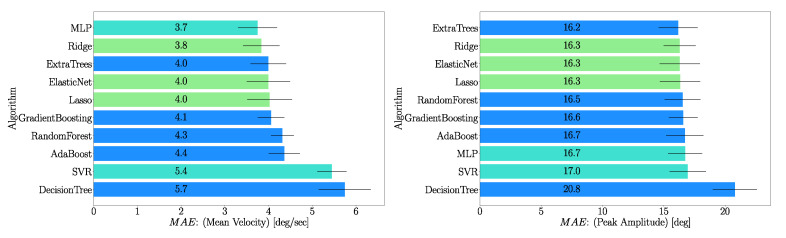
Comparison of MAE for mean velocity and peak amplitude using all features ($n_{kp}=10,\theta_{c}=5^{\circ }$). Features with top 20% of MI scores are used here for model training. Algorithms based on decision trees technique are color coded in blue, least squares in green and turquoise for Support Vector Regression (SVR) and Multiple Layer Perceptron (MLP).

A comparison between regularization algorithms selected for this research is shown in Figure 11. This comparison was done for $n_{kp}=10,\theta_{c}=5^{\circ }$. We can see that the MLP algorithm is the best performing algorithm when predicting mean velocity, whereas Extra Trees is the best one for predicting the peak amplitude. However, for the prediction of the peak amplitude, all algorithms seem to perform similarly with the exception of Decision Tree, which is the worst performing one for both inference parameters.

### 3.4. Model Prediction Performance

**Figure 12:**
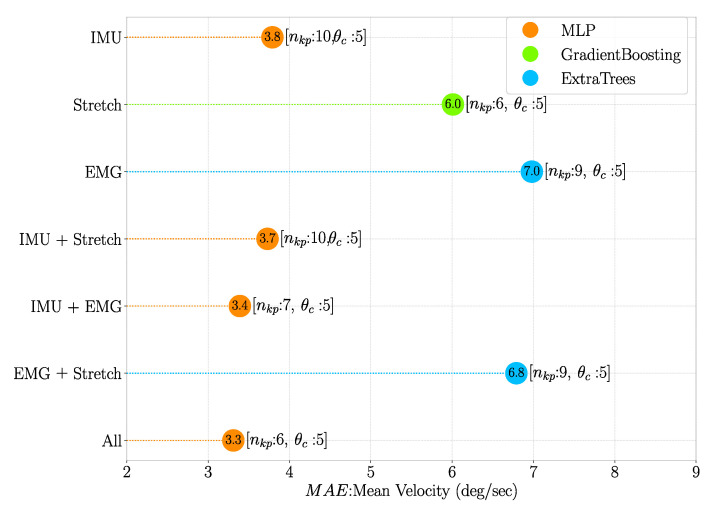
Mean Velocity MAE for the different features combinations, where the top 20% most important features according to their MI scores were used. The $n_{kp}$ and $\theta_{c}$ values at which minimum MAE was obtained are shown within square brackets.

**Figure 13:**
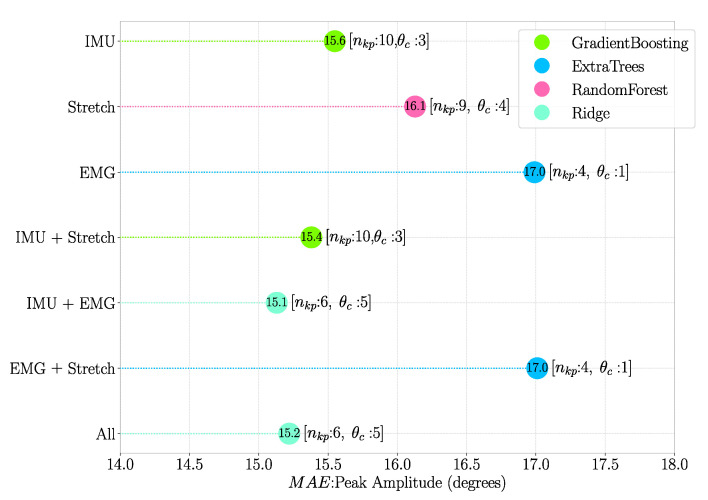
Peak amplitude MAE for the different features combinations, where the top 20% most important features according to their MI scores were used. The $n_{kp}$ and $\theta_{c}$ values at which minimum MAE was obtained are shown within square brackets.

Model prediction performance was evaluated iteratively for all possible combinations of the 10 selected algorithms, $n_{kp}$ and $\theta_{c}$, where $n_{kp}\in \left[1,10\right]$ and $\theta_{c}\in {\left[1,5\right]}^{\circ }$. The combination for which MAE values were minimum is shown in Figure 12 and Figure 13 for mean velocity and peak amplitude, respectively.

## 4. Discussion

In this paper, we used a multi-modal wearable sensor system to predict the elbow flexion trajectories of the upper limb. Through evaluation and comparison of MI and MAE values, the suitable features and algorithms were investigated to achieve accurate prediction of elbow trajectories.

It can be seen from Figure 7 that as $n_{kp}$ increases, the MAE of the predictions also increases, peaking for both mean velocity and peak amplitude at $n_{kp}\approx 75$. For $n_{kp}>75$, MAE decreases and plateaus for MAE values larger than the values observed for approximately $n_{kp}<50$. The reason behind the occurrence of a peak in the MAE for a certain $n_{kp}$ is unclear. However, the predictions with the lowest errors occur for lower $n_{kp}$ values, therefore we concluded such occurrence to be of minor importance. Using a small number of data points for making predictions can contribute to faster computation times, which is a fundamental requirement in real-time predictive systems. Therefore, it is recommended to use a low number of data points.

Regarding the length of the OW, the prediction error of MAE for mean velocity decreases as $\theta_{c}$ increases. This is not surprising, as a larger OW corresponds to a longer duration for extracting features and hence more information regarding intention of the subject is embedded in the signal. However, such a result is not so evident when considering peak amplitude, as the largest difference in MAE for the different possible $\theta_{c}$ was approximately 1${}^{\circ }$. Considering that the inference error for peak velocity only increases about 0.5 deg/s when using $\theta_{c}=3^{\circ }$ rather than $\theta_{c}=5^{\circ }$, the choice of $\theta_{c}=3^{\circ }$ could be a beneficial one, depending on the priority of the model. If the priority lies in achieving the best possible performance, then it is clear that an OW with $\theta_{c}$ larger than $3^{\circ }$ is preferable. In contrast, if the priority is in predicting the elbow trajectory as early as possible, without having a significant loss of accuracy, then using $\theta_{c}=3^{\circ }$ is a viable option.

**Figure 14:**
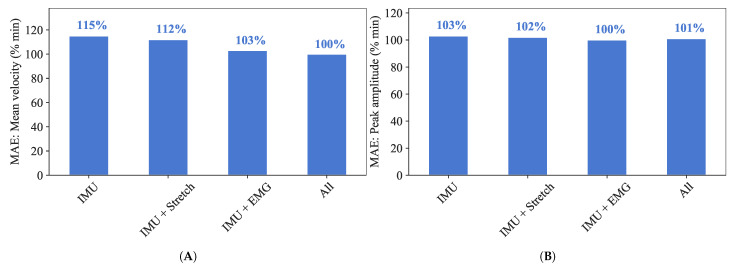
Best four combinations of sensors, features, $n_{kp}$, $\theta_{c}$ and algorithm in terms of percentage of the minimum observed MAE value for (**A**) mean velocity and (**B**) peak amplitude.

Features obtained from IMU sensor data seem to be the most important ones for predicting the defined inference parameters. This is extremely evident when considering the mean velocity, with the top 20% most important features being all obtained through IMU sensors (Figure 9A). Although not as dominant in the case of peak amplitude (Figure 9B), IMU features are still overall the ones most correlated with this inference parameter. This is likely because the inference parameters represent kinematic variables, and IMUs provide kinematic features only. Stretch sensor features also seem to be important, especially for the prediction of peak amplitude, but far less correlated than IMU based features. This suggests that kinematic signals are more relevant than physiological ones when it comes to predicting defined inference parameters, and, in truth, EMG-based features play little to no role. This can also be seen in Figure 12 and Figure 13, where there is a minor increase in the error of prediction when only kinematic features (Only IMU) are considered vs. IMU + stretch, IMU + EMG or all types of features considered (Figure 14). Such results lead us to think that for this particular type of elbow trajectories, using a single type of sensors—kinematic—should suffice to achieve satisfactory motion intention prediction. The slight improvement that would come from considering physiological sensors as well is outweighed by the decreased complexity that results from using only kinematic sensors: there are fewer used sensors, making the sensing system overall simpler to equip, and the computations performed by the model simpler as well.

The algorithm that seems to perform the best in predicting mean velocity is MLP, as it was the one which resulted in the lowest errors using all features for a certain $n_{kp},\theta_{c}$ (Figure 11) and also when investigating all different combinations of algorithms, $n_{kp}$ and $\theta_{c}$ (Figure 12). Although MLP was not present in the best combinations when predicting peak amplitude with different sets of features (Figure 13), one can see from Figure 11 that the difference in performance between different algorithms seems to be negligible. One of the reasons why MLP seems to perform well is probably due to the fact it is based on neural networks, which are known for having good performance when there is a small number of features with high correlation with the predicted variables (note that we chose the 20% top performing features) [40]. Another algorithm that seems to have performed well is Ridge, which could be an equally valid final choice. However, even though computation times were not investigated in this research, the authors are aware that such a characteristic plays an important role in the choice of algorithm. If the model is to be implemented with the aim of real-time prediction, MLP could prove to be a less optimal choice, as neural network-based approaches are often associated with longer computation times. On the other hand, regularisation-based algorithms are not computationally intensive, so they could prove to be the best option in that case.

This comparative analysis can provide several useful suggestions. First, this study provides a strategy for properly choosing OW and $n_{kp}$. Second, features from IMU sensor data achieved better performance of prediction comparing with other sensory features. In general, EMG signals are more widely used for human MID [41], but our results suggest that models using solely EMG data do not perform as well has ones using IMU sensor data for MID of elbow flexion. Third, the MLP algorithm was observed to result in good performance when inferring mean velocity, and the Ridge or Gradient Boosting algorithms for inferring peak amplitude. It is important to emphasize that the framework for developing MID algorithms mainly include three aforementioned guidelines. However, the strategy taken should vary depending on different application scenarios. For example, in the field of prosthetic engineering, subjects can appropriately eliminate the number of EMGs and decrease OW and keypoints under the condition of ensuring the control accuracy. A less complicated wearable sensor system can enhance the comfort and convenience of the subject.

There are still some limitations and challenges that need to be addressed. This study discusses single joint movement only, which does not encompass all movements performed by the upper limb during daily life activities. In future studies, we intend to explore the performance of our models by collecting data for more complex motions, such as planar reach and grasp. As a next step, we intend to implement an HMI with an upper limb assistive robot. The MID capability of this HMI will be adjusted according to the findings of the study here presented, namely in the choice of sensors and algorithm to be used. The performance of the HMI will then be evaluated in a scenario where the user’s upper extremity is being assisted. The findings also can be explored in the prostheses technology. The multimodal wearable system can detect and fuse user’s motion intention as input to control the prostheses devices.

## 5. Conclusions

In this study, a multimodal wearable sensor system was used to extract features from kinematic and physiological signals acquired during the performance of elbow flexion movements. We analysed the impact of selecting different groups of features and various algorithms. To the best of the authors’ knowledge, this is the first multimodal wearable upper limb sensing system that uses signals acquired from EMG, IMU and stretch sensors. We have concluded that, depending on the priority of the developer of the system, there are different combinations of sensors that should be used. If one prefers to have a system that can quickly detect motion intention at the cost of a slight decrease in accuracy, using kinematic signals only and with a small observation window is a preferred option. If instead, the priority is on having the best possible prediction, a longer observation window and using both physiological and kinematic features is necessary. However, such a decision implies the use of extra sensors for detecting muscle activity, which can increase the complexity of the system.

## Figures and Tables

**Table 1:** MAE for all features and top 20% of most important features according to the MI scores. Values are represented as mean value ± standard deviation.

Parameter	MAE	
	All Features	Top 20%
Mean Velocity	4.78 ± 0.47	4.34 ± 0.40
Peak Amplitude	17.56 ± 1.62	16.87 ± 1.50

## Data Availability

Data available on request.

## Abbreviations

The following abbreviations are used in this manuscript:

AD	Anterior Deltoid
BB	Biceps Brachii
EEG	Electroencefalography
sEMG	Surface Electromyography
HMI	Human-machine interface
IMU	Inertial measurement unit
MAE	Mean absolute error
MI	Mutual information
MID	Motion intention detection
MJT	Minimum jerk trajectory
MLP	Multiple Layer Perceptron
MMG	Mechanomyographic
OW	Observation window
PM	Pectoralis Major
SVR	Support Vector Regression
TB	Triceps Brachii
